# Sigma-2 receptor ligands potentiate conventional chemotherapies and improve survival in models of pancreatic adenocarcinoma

**DOI:** 10.1186/1479-5876-7-24

**Published:** 2009-03-26

**Authors:** Hiroyuki Kashiwagi, Jonathan E McDunn, Peter O Simon, Peter S Goedegebuure, Suwanna Vangveravong, Katherine Chang, Richard S Hotchkiss, Robert H Mach, William G Hawkins

**Affiliations:** 1 Department of Surgery, Washington University School of Medicine, 660 S. Euclid Avenue, Campus Box 8109, St. Louis, MO 63110, USA; 2 Department of Anesthesiology, Washington University School of Medicine, 660 S. Euclid Avenue, St. Louis, MO 63110, USA; 3 Alvin J. Siteman Cancer Center, Washington University School of Medicine, 660 S. Euclid Avenue, Campus Box 8109, St. Louis, MO 63110, USA; 4 Department of Radiology, Washington University School of Medicine, 660 S. Euclid Avenue, St. Louis, MO 63110, USA

## Abstract

**Background:**

We have previously reported that the sigma-2 receptor is highly expressed in pancreas cancer. Furthermore, we have demonstrated that sigma-2 receptor specific ligands induce apoptosis in a dose-dependent fashion. Here, we examined whether sigma-2 receptor ligands potentiate conventional chemotherapies such as gemcitabine and paclitaxel.

**Methods:**

Mouse (Panc-02) and human (CFPAC-1, Panc-1, AsPC-1) pancreas cancer cell lines were used in this study. Apoptosis was determined by FACS or immunohistochemical analysis after TUNEL and Caspase-3 staining. Combination therapy with the sigma-2 ligand SV119 and the conventional chemotherapies gemcitabine and paclitaxel was evaluated in an allogenic animal model of pancreas cancer.

**Results:**

SV119, gemcitabine, and paclitaxel induced apoptosis in a dose-dependent fashion in all pancreas cancer cell lines tested. Combinations demonstrated increases in apoptosis. Mice were treated with SV119 (1 mg/day) which was administered in combination with paclitaxel (300 μg/day) over 7 days to mice with established tumors. A survival benefit was observed with combination therapy (p = 0.0002). Every other day treatment of SV119 (1 mg/day) in combination with weekly treatment of gemcitabine (1.5 mg/week) for 2 weeks also showed a survival benefit (p = 0.046). Animals tolerated the combination therapy and no gross toxicity was noted in serum biochemistry data or on necropsy.

**Conclusion:**

SV119 augments tumoricidal activity of paclitaxel and gemcitabine without major side effects. These results highlight the potential utility of the sigma-2 ligand as an adjuvant treatment in pancreas cancer.

## Background

Pancreas cancer is the fourth leading cause of cancer-related mortality in the United States [1]. The 5-year survival rate is less than 5% [2]. This poor outcome stems from the difficulty in achieving an early diagnosis and the failure of surgery, radiation and chemotherapy. In fact, only 15% of patients are eligible for surgical resection at the time of diagnosis [3]. Even after radical pancreatectomy, most patients with pancreas cancer show local recurrence or metastasis within 1 year. The current standard chemotherapeutic, gemcitabine, demonstrates a slight improvement in survival, but these modest results are not satisfactory [4]. Novel therapeutic strategies are desperately needed.

Standard therapies for pancreatic cancer have two major limitations. First, systemic administration of chemotherapy does not selectively target the cancer and is limited by systemic toxicity. Second, local therapies such as radiation or surgery do not address the potential for distant metastases. For these reasons, a targeted strategy which directly delivers the cytotoxic molecule to the cancer is highly desirable.

There is considerable interest in stimulating apoptosis and inhibiting survival machinery as components of cancer therapy [4-6]. Many oncogenic transformations result from the inactivation or deletion of pro-apoptotic genes or the translocation of an anti-apoptotic gene downstream of highly active promoters [5,7,8]. The sigma-2 receptor is a unique targeting receptor that induces tumor apoptosis for pancreas cancer. The sigma receptor was initially proposed as a subtype of opioid receptors [9]. Early receptor binding studies using benzomorphan opioids indicated at least two subtypes of sigma receptors exist: sigma-1 and sigma-2 subtype [5]. These subtypes display different tissue distributions and distinct physiological and pharmacological profiles in both the central and peripheral nervous systems. Although natural ligands for these receptors are still unknown, recent research has demonstrated that sigma receptors are over-expressed in a variety of human and rodent tumors [5,6,10,11] and that synthetic ligands to this receptor could play an important role in cancer diagnosis and therapy [12]. We have previously reported that the sigma-2 receptor is highly expressed in pancreas cancer and weakly expressed in normal pancreas [13]. In this same study, we carefully characterized the receptor-ligand binding interaction and reported the *K*d and *B*max values of sigma-2 receptor ligands in models of pancreatic adenocarcinoma. Furthermore, we have demonstrated that sigma-2 receptor specific ligands induce apoptosis in a dose-dependent fashion and that this activity occurs, at least in part, via the intrinsic apoptotic pathway. Because sigma-2 receptor-specific ligands selectively induce apoptosis in pancreas cancer, these ligands may act as sensitizers to standard chemotherapies.

Since pancreatic cancer has proven to be resistant to modern, conventional therapies, we have chosen to focus our efforts and developing novel therapeutics that specifically target this cancer. In this study, we follow up on our previous characterization of sigma-2 receptor ligands by demonstrating that these novel agents augment conventional therapies for pancreas cancer and are an exciting class of compounds for potential treatment of these malignancies.

## Methods

### Sigma receptor ligands

Sigma-2 specific ligands SV119, SV95, and fluorescent -labeled sigma-2 ligand, SW120, were synthesized and prepared as previously described [13-15]. The Sigma-1 receptor ligand, (+)-pentazocine (Sigma Chemical, St. Louis, MO), was used as a control.

### Cell lines

Murine pancreatic adenocarcinoma, Panc-02, was obtained from Bryan Clary (Duke University) and maintained in supplemented RPMI 1640 containing glutamine (2 mmol/L), pyruvate (1 mmol/L), penicillin (100 IU/mL), streptomycin (100 IU/mL), and 10% FBS. Human pancreatic adenocarcinoma cell lines (Panc-1, AsPC-1, and CFPAC-1) were obtained from ATCC (Bethesda, MD) and maintained in Dulbecco's modified eagle's medium (DMEM) containing glutamine (2 mmol/L), pyruvate (1 mmol/L), penicillin (100 IU/mL), streptomycin (100 IU/mL), and 10% FBS. HPDE (Human Pancreas Duct Epithelium) was obtained from Dr. Ming Sound Tsao and cultured in Keratinocyte serum-free (KSF) medium (Gibco/Invitrogen, Carlsbad, CA) with 50 mg/ml bovine pituitary extract (BPE), 5 ng/ml epidermal growth factor (EGF), and 1× antibiotic-antimycotic cocktail (Gibco/Invitrogen). All cell culture processes were carried out in a humidified atmosphere of 5% CO_2 _at 37°C. All cultures were free of *Mycoplasma *as assayed by the Washington University Division of Comparative Medicine. Cultures were maintained for no longer than 12 weeks after recovery from frozen stocks.

### Sigma-2 ligand binding *in vitro*

Tumor cells were incubated with 10 nM of SW120 (a fluorescent-labeled sigma-2 receptor ligand) for 30 minutes. HPDE cells were used as a normal control. To demonstrate the specificity of SW120 for Sigma-2 receptor binding, 10μM of SV95 (Sigma-2 ligand) or (+)-pentazocine (sigma-1 receptor ligand) were added to cells 30 minutes prior to SW120 treatment. All lines were then washed 3 times with PBS and evaluated by flow cytometry.

### Evaluation of cytotoxicity *in vitro*

Tumor cells were harvested and seeded at a density of approximately 0.2 × 10^6 ^cells per well in 12-well plates in 1.0 ml culture medium. Seeded cells were split and pre-incubated for more than 24 hours (Panc-02) and 48 hours (CFPAC-1, AsPC-1, and Panc-1) to maintain their growth conditions. SV119 and SW120 were dissolved in DMSO, and gemcitabine and paclitaxel were dissolved in PBS. The solutions were then added to the culture medium at the concentrations indicated with final concentration of DMSO at less than 1%. The extent of apoptosis was subsequently measured as previously described [13]. Briefly, staining was performed on trypsin-EDTA treated cultures fixed with 1% paraformaldehyde and 90% methanol. Fixed cells were resuspended in TUNEL reagent or cleaved caspase-3 antibody and incubated overnight at room temperature (TUNEL) or 4°C (Caspase 3). After incubated cells were washed, cells were resuspended in fluorescent antibody or 7-AAD buffer and incubated for 1 hour at room temperature. Cell-associated fluorescence was determined by the flow cytometry (FACScan, BD Biosciences) and analyzed with CellQuest software (BD Biosciences).

### *In vivo *assessment of apoptosis

Female C57BL/6 mice (8–12 weeks old) were purchased from the NCI and acclimated for at least 1 week before tumor implantation. All mice were injected in the right flank with 200 μl single cell suspension containing 1.0 × 10^6 ^Panc-02 cells. Two weeks after tumor implantation, at which point the mean tumor diameter was approximately 5 mm, mice were treated with a single intraperitoneal injection of SV119, conventional chemotherapy, or both. Twenty-four hours later, tumors were harvested and minced to 1 mm and digested in a RPMI buffer containing 1 mg/ml collagenase (Sigma-Aldrich, St. Louis, MO) and 0.1 mg/ml DNase (Sigma-Aldrich, St. Louis, MO) for 45 min to obtain a single-cell suspension. After filtering, erythrocyte contaminants were lysed in Ammonium Chloride (ACK) buffer, pelleted, and resuspended in PBS (pH 7.4). Single cell suspensions were fixed by 1% paraformaldehyde by following the above procedure. Apoptosis was then assessed as described above utilizing flow cytometry.

### *In vivo *assessment of tumor growth and survival

Female C57BL/6 mice (8–12 weeks old) were purchased from the NCI and acclimated for at least 1 week before tumor implantation. All mice were injected in the right flank with 200 μl single cell suspension containing 1.0 × 10^6 ^Panc-02 cells. Treatment of tumors started 2 weeks after tumor implantation, at which point the mean tumor diameter was approximately 5 mm. To evaluate the effect of treatment both systemically and on tumors *in vivo*, several treated mice were sacrificed and blood cytologic (complete blood count) and biochemical analysis (liver enzymes, bilirubin, amylase, lipase, BUN, creatinine, glucose) were performed. For the survival study, tumor bearing mice (n = 8–10 per group) were treated with SV119 and/or chemotherapy once daily for 7 days (paclitaxel treatment model) or every other day for 14 days (gemcitabine treatment model). Mean tumor diameter was measured three times each week. All mice were euthanized when their tumor ulcerated, reached a mean diameter of 15 mm, or 50 days after initiation of the study. All studies were performed in accordance with an animal protocol approved by the Washington University Institutional Animal Care Facility.

### Statistical analysis

Error bars, unless stated otherwise, represent means plus or minus SEM of an experiment with at least three biological replicates. For statistical analysis of differences between groups, one-way ANOVA was performed. For *in vivo *experiments, Kaplan-Meier survival curves were plotted and differences were compared with a log-rank test. A *p*-value less than 0.05 was considered significant for all analysis.

## Results

### Sigma-2 ligands have a high affinity for pancreatic adenocarcinoma cell lines compared to normal cell lines

**Figure 1:**
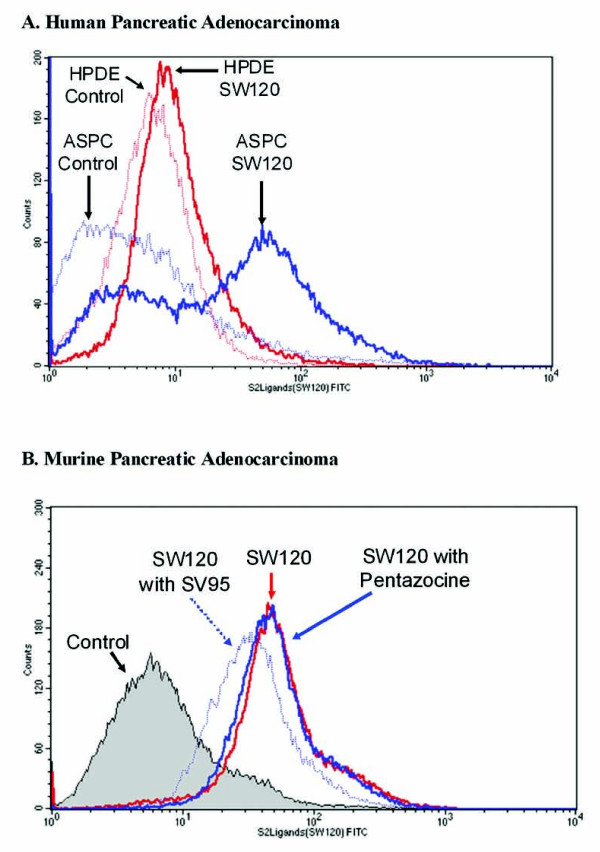
**Sigma-2 ligands have a high affinity for pancreatic adenocarcinoma cell lines compared to normal cell lines**. Representative FACS analysis of human (A.) and murine (B.) pancreatic adenocarcinoma cell lines treated with the FITC-conjugated Sigma-2 ligand, SW120. In Panel A, HPDE (immortalized pancreatic ductal epithelial cells) were used as a control. In Panel B, competitive inhibition of SW120 binding was demonstrated by preincubation with the Sigma-2 ligand, SW95. Pentazocine, a Sigma-1 receptor ligand, was also used as a control and did not demonstrate competitive inhibition. Experiments were performed in triplicate with comparable results.

We have previously reported that murine (Panc-02) and human (AsPC-1, CFPAC-1, and Panc-1) pancreatic adenocarcinoma cell lines display increased expression of the sigma-2 receptor [13]. However, we have not previously compared the binding of Sigma-2 ligands to the normal human pancreas cell line HPDE. As demonstrated in Figure 1, Panel A, there is a high affinity of Sigma-2 ligand to the human pancreatic adenocarcinoma cell line AsPC-1 compared to the immortalized normal pancreatic cell line HPDE. This binding also appeared to be specific to the Sigma-2 receptor as we were able to demonstrate competitive inhibition by pretreating with a second Sigma-2 ligand, but not a Sigma-1 receptor ligand (pentazocine, Panel B)

### The apoptotic effect of the sigma-2 ligand, SV119, is enhanced by conventional chemotherapy *in vitro*

**Figure 2:**
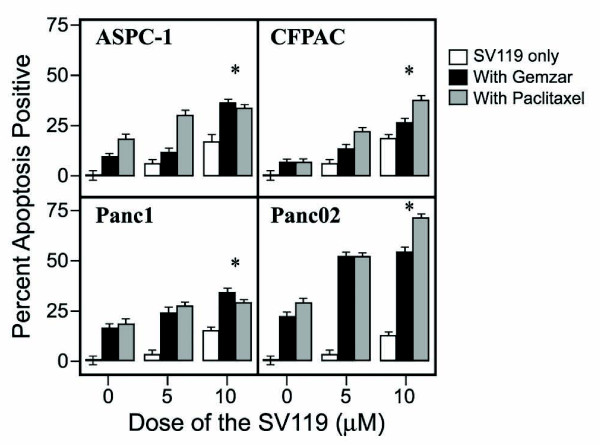
**The apoptotic effect of the sigma-2 ligand, SV119, is enhanced by conventional chemotherapy *in vitro***. Model pancreatic adenocarcinoma cell lines were treated with escalating doses of SV119, SV119 and gemcitabine, or SV119 and paclitaxel. After 24 hours of treatment, percent caspase-3 positive cells were determined by flow cytometry. Results are expressed as the mean, with bars representing standard error of the mean. Experiments were performed in triplicate with comparable results. Where indicated, * = *P *< 0.01 for SV119+gemcitabine or SV119+paclitaxel vs. SV119-only control.

In order to evaluate the potential therapeutic effect of the sigma-2 ligand, SV119, in combination with conventional chemotherapy, we treated pancreatic cancer cell lines with SV119 and the chemotherapeutic agents gemcitabine and paclitaxel. After 24 hours of treatment in the presence of SV119 and gemcitabine or paclitaxel, all cell lines demonstrated an additive increase in apoptosis as demonstrated by increases in TUNEL staining (Figure 2). Similar responses were noted in all cell lines when cleaved caspase 3 was utilized as the endpoint (data not shown).

### The sigma-2 ligand SV119 induces moderate apoptosis in both G0 and G1 to G2/S phase of pancreatic cancer cells *in vitro*

**Figure 3:**
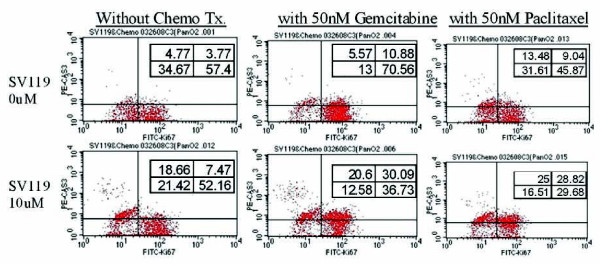
**The sigma-2 ligand SV119 induces moderate apoptosis in both G0 and G1 to G2/S phase of pancreatic cancer cells *in vitro***. The murine pancreatic adenocarcinoma cell, Panc02, was treated with SV119 alone or in combination with gemcitabine or paclitaxel. After 24 hours of treatment, samples were stained for cleaved caspase-3 and Ki67. Representative histograms are shown from an experiment performed in triplicate.

Next, in order to further characterize this effect, we evaluated the growth phase of these pancreatic cancer cells under these conditions by co-staining for cleaved caspase-3 and the proliferation maker Ki-67. As seen in Figure 3, SV119 and gemcitabine or paclitaxel induced apoptosis in cells that were both in G0 as well as in G1 to G2/S phase of the cell cycle. Mean TUNEL-positivity ranged from 16.1% to 18.6% at 10 μM SV119 (Figure 3). Combining SV119 with a chemotherapy increased apoptosis. Mean TUNEL-positivity ranged from 26.5% to 70.5% in the SV119 and gemcitabine combination (50 nM) and from 26.6% to 53.8% in the SV119 and paclitaxel combination (50 nM). As shown in the representative FACS histogram, SV119 (10 μM) induced moderate apoptosis in Ki67 negative cells (G0 phase). Gemcitabine treatment shifted the cell proliferation from G0 to the active stage with moderate apoptosis (Figure 3). Paclitaxel demonstrated limited apoptosis in both G0 and active phases of the cancer cell cycle. These data suggest that SV119 may serve as a sensitizer to these conventional therapies.

### The pro-apoptotic activity of the sigma-2 ligand, SV119, is enhanced by conventional chemotherapy *in vivo *without cytologic or chemical evidence of systemic toxicity

**Figure 4:**
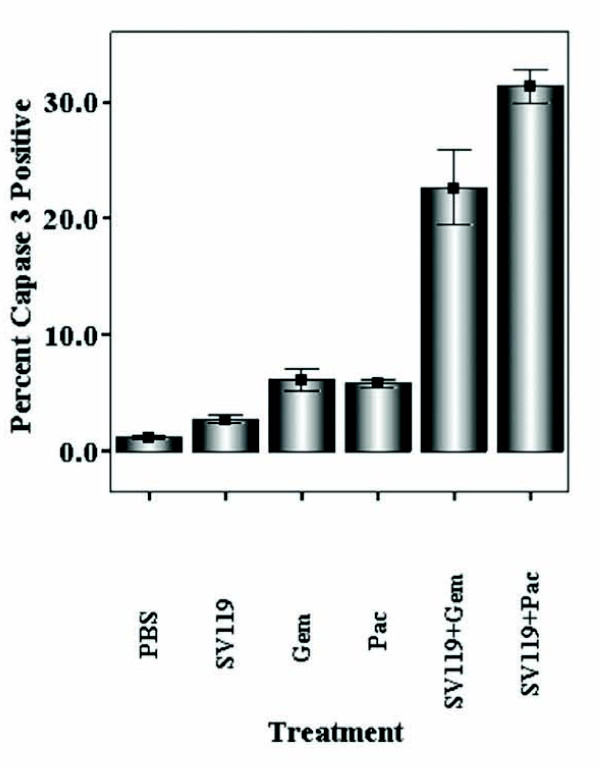
**The pro-apoptotic activity of the sigma-2 ligand, SV119, is enhanced by conventional chemotherapy *in vivo***. C57BL/6 mice bearing implanted tumor allografts were treated with a single dose of SV119 and conventional chemotherapy (gemcitabine or paclitaxel). Twenty-four hours after treatment, tumors were harvested and single cell suspensions were generated. Percent active caspase-3 was then measured in tumor cells by flow cytometry. Each experimental group represents an n = 3. Results are expressed as the mean, with bars representing standard error of the mean.

In order to determine if the pro-apoptotic effect of these agents was also conferred to tumors *in vivo*, an implantable murine tumor model was utilized. In this study, pancreatic tumors were implanted into the flank of C57BL/6 mice. Fourteen days after tumor implantation, a single intraperitoneal treatment on SV119, or SV119 combined with conventional chemotherapy (gemcitabine or paclitaxel) was administered. Twenty-four hours later, single cell suspensions of these tumors were generated and apoptosis was measured by FACS analysis. As shown in Figure 4, apoptosis was markedly increased in samples that were treated with both sigma-2 ligand (SV119) and conventional chemotherapy (gemcitabine or paclitaxel). These mice appeared healthy and cytologic/biochemical laboratory analysis did not reveal major toxicity (Additional file 1) [16]. Necropsy was also performed on selected animals and no gross or histologic evidence of organ dysfunction was observed (data not shown).

### Treatment of mice bearing pancreatic tumor allografts with the sigma-2 receptor ligand, SV119, and conventional chemotherapy slows tumor growth and confers a survival advantage

**Figure 5:**
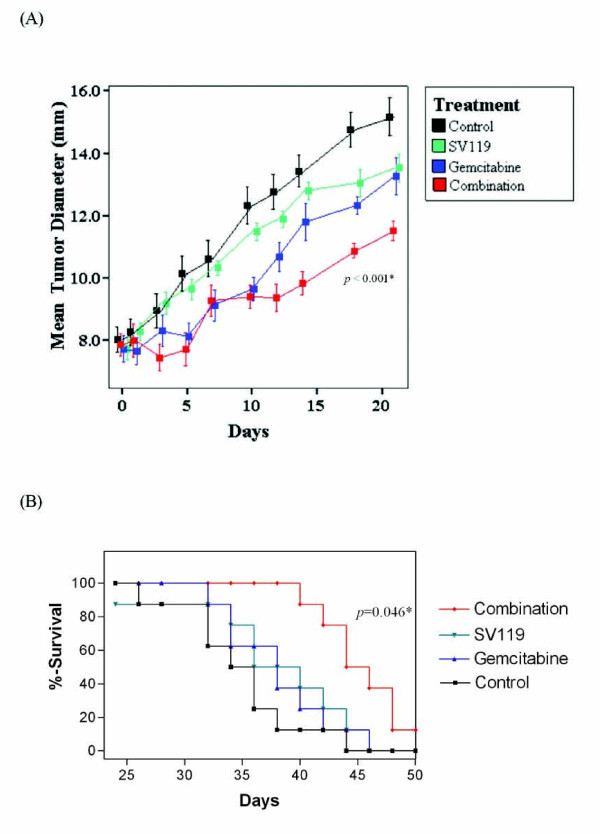
**The sigma-2 ligand, SV119, combined with gemcitabine suppresses tumor growth and increases survival in model pancreatic adenocarcinoma *in vivo***. C57BL/6 mice bearing established tumor allografts were treated with every other day SV119 (1 mg/mouse, i.p. for 7 days) and weekly gemcitabine (3 mg/mouse, i.p. for two weeks). Mean tumor diameter (Panel A) and survival (Panel B) were measured. * = vs. control.

**Figure 6:**
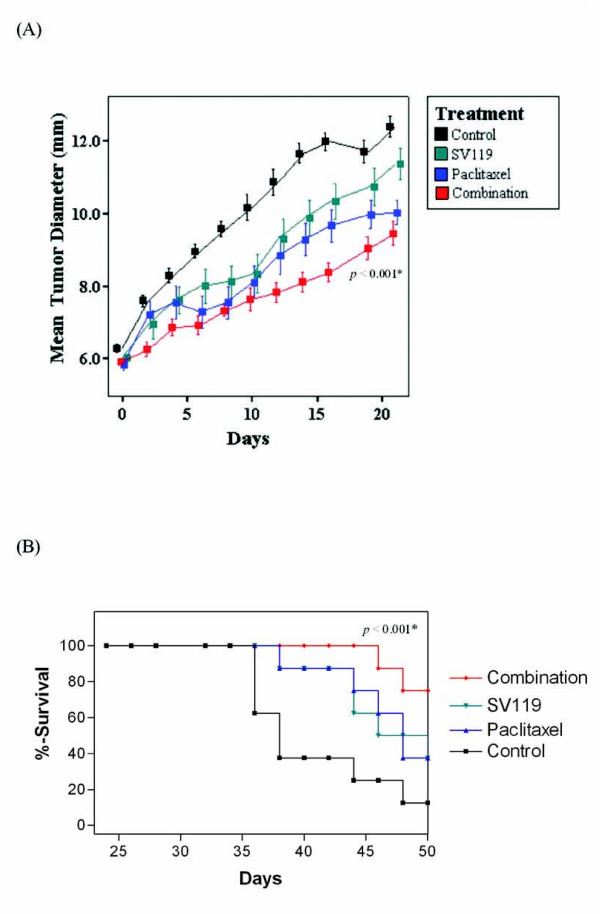
**The sigma-2 ligand, SV119, combined with paclitaxel suppresses tumor growth and increases survival in model pancreatic adenocarcinoma *in vivo***. C57BL/6 mice bearing established tumor allografts were treated with daily SV119 (1 mg/mouse, i.p. for 7 days) and daily paclitaxel (0.3 mg/mouse, i.p. for 7 days). Mean tumor diameter (Panel A) and survival (Panel B) were measured. * = vs. control.

Two different treatment models of SV119 in combination with conventional chemotherapies were utilized. In the first model, weekly treatment of gemcitabine (1.5 mg/week) in combination with every other day treatment of SV119 was given for 2 weeks (Figure 5). In the second model, paclitaxel (0.3 mg/day) and SV119 were used as concurrent daily treatments (Figure 6). A suboptimal dosing regimen was selected to maximize our chances of detecting a combined effect.

*In vivo *systemic administration of SV119-alone given as 7 daily doses or as 7 doses every other day for 14 days demonstrated a non significant tumor volume and survival advantage. Treatment with chemotherapies alone (gemcitabine or paclitaxel) also demonstrated a limited effect in both treatment models. However, in both models, the combination of SV119 with a chemotherapeutic agent significantly slowed tumor growth when compared to therapy with single agents or with untreated controls. Animals tolerated the combination therapy well, without evidence of cytologic or biochemical toxicity (data not shown).

## Discussion

Pancreas cancer remains a devastating malignancy and novel therapeutic strategies are desperately needed. Cancers by definition create and develop in a stressful environment (overcrowding, hypoxia, nutrient starvation) which should promote apoptosis. Therefore most cancers including pancreas cancer develop numerous strategies which promote survival and overcome natural signals to undergo apoptosis [17]. In fact, many experts suggest that suppression of apoptosis is central to the evolution of cancer. It is also an important factor for resistance to many standard cancer treatments [12,18-21]. Standard therapies including most chemotherapeutics and radiation therapy induce cellular stress and thereby promote apoptosis. Standard therapies capitalize on the premise that cells in stressful microenvironments have increasing susceptibility to apoptogenic stimuli when subjected to additional cellular stressors such as cytotoxic therapeutics. This argument appears to be true for the common therapeutics utilized in the treatment of pancreas cancer. For example gemcitabine inhibits DNA replication, indirectly promoting apoptosis, and paclitaxel arrests the cell cycle, directly promoting apoptosis.

The sigma-2 receptor and its undiscovered endogenous ligand(s) is poorly understood. Literature regarding the role of the sigma-2 receptor in normal homeostasis is unquestionably lacking. Most of what is understood about this receptor comes from investigations in tumors. Several groups of investigators have shown that sigma-2 receptor expression is markedly increased across diverse malignancies. Recent data have suggested that this upregulation is related to cell proliferation [5]. This feature has generated interest in utilizing sigma-2 ligands as radiotracers for cancer imaging. Our group has shown that once the receptor is engaged certain ligands are rapidly internalized and distributed to membrane-encapsulated organelles [11]. This finding is consistent with the report by Ostenfeld et al that siramesine, a sigma-2 receptor selective ligand, is lysosomotrophic [15].

We and others have recently shown that selected sigma-2 ligands are capable of inducing apoptosis in a multitude of human and murine cancer cells lines and in animal models of cancer including pancreas cancer [12,13,15]. The mechanisms by which this works are poorly understood but we do know that the apoptosis generated by selected sigma-2 ligands can be partially inhibited with intrinsic pathway inhibitors like caspase inhibitor [13]. While the anti-tumor effects of sigma-2 ligands alone are modest, the high receptor abundance on cancers and the high affinity of the ligands for the receptor may present a unique opportunity to utilize these ligands as chemotherapeutic sensitizers.

We hypothesized that sigma-2 ligands may selectively augment the effects of non-selective pro-apoptotic anti-cancer therapies preferentially in cancer cells. The high tumor receptor abundance may provide a novel strategy for improving on the effects of cytotoxic chemotherapies without increasing toxicity. Since sigma-2 ligands are expressed on other tissues (although at lower levels) we were concerned that such a combined strategy might result in toxicity wherever sigma-2 ligands are found. We tested whether SV119 (an apoptogenic sigma-2 ligand) and a standard chemotherapeutic would slow tumor growth, reduce toxicity, and ultimately improve survival in a murine model of established pancreas adenocarcinoma.

In our present study, both the specific ligand of the Sigma-2 receptor (SV119) and the chemotherapies showed moderate apoptosis in all pancreas cancer cells *in vitro*. SV119 induced tumor apoptosis in both cycling cells at all phases (G1 to G2M/S) and in quiescent, G0, cells (Figure 3). Depending on the cell line assayed, SV119 in combination with the lower dose of chemotherapies showed an additive or super-additive effect in inducing tumor apoptosis (Figure 3). These results indicate that SV119 is a useful sensitizer for pancreas cancer treatment in combination with cell cycle specific chemotherapies. In addition, the combination of SV119 with standard chemotherapy may decrease the chemotherapy dose required. This is significant because it is typically the systemic toxicity of contemporary chemotherapeutics that limit their effectiveness.

In the allograft C57/BL6 model of pancreas cancer, SV119 treatment in combination with gemcitabine or paclitaxel led to tumor stability and regression in some cases when compared to single therapies. Although all tumors resumed growing shortly after treatment was stopped, tumors in mice receiving combination treatment grew more slowly than tumors in either of the single agent treatments or vehicle-injected control. This result suggests that combination therapy was not only successful in reducing tumor mass but also altered the course of tumor growth after therapy was stopped. Importantly no significant toxicities were appreciated by serum biochemistry or by necropsy and immunohistochemistry.

## Conclusion

Pancreas cancer is an aggressive and rapidly metastasizing tumor and we believe that it is unlikely that a single therapeutic will result in a cure for this devastating cancer. Here, we have demonstrated that the sigma-2 receptor-specific ligand, SV-119, potentiates cell death when combined with conventional chemotherapies without appreciable toxicity in model pancreatic adenocarcinoma. It is highly critical to investigate novel strategies which might complement or enhance other proven anti-cancer regimens for the treatment of pancreas cancer. We believe that this experimental design highlights a new potential strategy for the treatment of pancreas cancer and warrants further exploration.

## Competing interests

The authors declare that they have no competing interests.

## Authors' contributions

HK Performed experiments, interpreted results, drafted manuscript. JEM Drafted manuscript, critical revision to manuscript, designed experiments, interpreted results. POS Drafted manuscript, critical revision to manuscript, designed experiments, interpreted results. PSG Critical revision to manuscript. SV Designed and conducted experiments. KC Designed and conducted experiments. RSH Critical revision to manuscript, designed experiments, interpreted results. RHM Synthesis of sigma-2 ligands, critical revision to manuscript. WGH Designed experiments, interpreted results, final draft of manuscript. All authors have read and approved the final manuscript.

## Supplementary Material

Additional file 1 **Table S1 – Serum toxicology and cytology of mice treated with the sigma-2 ligand, SV119, and conventional chemotherapy.** Peripheral blood was drawn from tumor-bearing mice 24 hours after treatment with a single dose of SV119 and conventional chemotherapy (gemcitabine or paclitaxel). Cytologic and serum chemistry evaluations were performed by the animal care facility at Washington University. Data is expressed as mean +/- standard error of the mean. Each experimental group represents an n = 2. Click here for file
