# Supplementary material for: Sigma-2 receptor ligands potentiate conventional chemotherapies and improve survival in models of pancreatic adenocarcinoma
Source: J Transl Med. 2009 Mar 26;7:24. doi: 10.1186/1479-5876-7-24 (PMC2669042; doi:10.1186/1479-5876-7-24)
Supplement: Additional file 1 — Table S1 – Serum toxicology and cytology of mice treated with the sigma-2 ligand, SV119, and conventional chemotherapy. Peripheral blood was drawn from tumor-bearing mice 24 hours after treatment with a single dose of SV119 and conventional chemotherapy (gemcitabine or paclitaxel). Cytologic and serum chemistry evaluations were performed by the animal care facility at Washington University. Data is expressed as mean +/- standard error of the mean. Each experimental group represents an n = 2. [file 1479-5876-7-24-S1.doc]

**Table S1 -** Serum toxicology and cytology of mice treated with the sigma-2 ligand, SV119, and conventional chemotherapy

|  | WBC (x10^3^/mm^3^) | RBC (x10^3^/mm^3^) | Hgb (g/dl) | PLT (x10^3^/mm^3^) |  |  |  |  |
| --- | --- | --- | --- | --- | --- | --- | --- | --- |
| Reference Range (16) | 2.8 +/- 0.8 | 9.42 +/- 0.3 | 13.9 +/- 0.3 | 8.9 +/- 1.3 |  |  |  |  |
| PBS | 3.81 +/- 2.1 | 8.47 +/- 1.4 | 12.3 +/- 1.7 | 724.7 +/- 403.5 |  |  |  |  |
| SV119 | 4.17 +/-0.2 | 8.38 +/- 0.02 | 12.3 +/- 0.5 | 864.5 +/- 224.2 |  |  |  |  |
| Gemcitabine | 2.55 +/- 1.2 | 8.92 +/- 0.3 | 12.7 +/- 0.4 | 901 +/- 93.2 |  |  |  |  |
| Gemcitabine + SV119 | 2.85 +/- 0.4 | 9.0 +/- 2.3 | 13.2 +/- 3.4 | 916.7 +/- 150.8 |  |  |  |  |
| Paclitaxel | 3.15 +/- 1.8 | 8.85 +/- 0.1 | 12.9 +/- 0.3 | 892.7 +/- 88.2 |  |  |  |  |
| Paclitaxel + SV119 | 2.49 +/- 0.7 | 8.65 +/- 0.02 | 12.3 +/-0.3 | 892.7 +/- 104 |  |  |  |  |
| p-value | NS | NS | NS | NS |  |  |  |  |
|  | ALT (U/l) | AST (U/l) | BUN(mg/dl) | CRE (mg/dl) | GLU (mg/dl) | Amylase (U/l) | Lipase (U/l) | TBIL (mg/dl) |
| Reference Range (16) | 40 +/- 18 | 91 +/- 33 | 31 +/- 2.5 | 0.8 +/- 0.3 | 206.9 +/- 29 | 2308 +/- 1538 | 96 +/- 348 | 0.2 +/- 0.1 |
| PBS | 69.0 +/- 41.0 | 28.3 +/- 4.2 | 15.8 +/- 5.2 | 0.43 +/- 0.1 | 260 +/- 51.7 | 1500.8 +/- 845.1 | 1029.5 +/- 57.3 | 0.3 +/- 0.2 |
| SV119 | 83.3 +/- 66.7 | 29.0 +/- 6.2 | 14.0 +/- 3.0 | 0.47 +/- 0.1 | 241 +/- 27.9 | 1490.7 +/- 585 | 1137.7 +/- 51.3 | 0.6 +/- 0.7 |
| Gemcitabine | 78.0 +/-40.0 | 38.0 +/- 21.0 | 16 +/- 3.0 | 0.4 | 268.3 +/- 32.5 | 1824.7 +/- 155 | 1049 +/- 108.9 | 0.3 +/- 0.3 |
| Gemcitabine + SV119 | 53.3 +/- 28.7 | 29.0 +/- 7.9 | 17.3 +/- 4.9 | 0.4 +/- 0.1 | 197.3 +/- 34.1 | 1453.3 +/- 160 | 930 +/- 116.8 | 0.3 +/- 0.3 |
| Paclitaxel | 76.7 +/- 28 | 33.3 +/- 3.5 | 15.7 +/- 2.9 | 0.4 | 247 +/- 4 | 2088.7 +/- 279 | 1015.3 +/- 42.5 | 0.2 +/- 0.1 |
| Paclitaxel + SV119 | 53.7 +/- 32.1 | 33.6 +/- 9.1 | 15.3 +/- 3.1 | 0.37 +/- 0.1 | 236 +/- 65.1 | 2077.3 +/- 512 | 1082.3 +/- 43.8 | 0.3 +/- 0.2 |
| p-value | NS | NS | NS | NS | NS | NS | NS | NS |
